# Sex differences in noise-induced hearing loss: a cross-sectional study in China

**DOI:** 10.1186/s13293-021-00369-0

**Published:** 2021-03-06

**Authors:** Qixuan Wang, Xueling Wang, Lu Yang, Kun Han, Zhiwu Huang, Hao Wu

**Affiliations:** 1 Department of Otolaryngology-Head and Neck Surgery, Shanghai Ninth People’s Hospital, Shanghai Jiao Tong University School of Medicine, 639 ZhiZaoJu Road, HuangPu District, Shanghai, 200011 People’s Republic of China; 2 Ear Institute, Shanghai Jiao Tong University School of Medicine, Shanghai, 200011 People’s Republic of China; 3 Key Laboratory of Translational Medicine on Ear and Nose Diseases, Shanghai, 200011 People’s Republic of China; 4 Biobank, Shanghai Ninth People’s Hospital, Shanghai Jiao Tong University School of Medicine, Shanghai, 200011 People’s Republic of China

**Keywords:** Sex differences, Occupational noise exposure, Hearing loss

## Abstract

**Background:**

Significant sex differences exist in hearing physiology, while few human studies have investigated sex differences in noise-induced hearing loss (NIHL), and the sex bias in previous studies resulted in inadequate female data. The study aims to investigate sex differences in the characteristics of NIHL to provide insight into sex-specific risk factors, prevention strategies and treatment for NIHL.

**Methods:**

This cross-sectional study included 2280 industrial noise-exposed shipyard workers (1140 males and 1140 females matched for age, job and employment length) in China. Individual noise exposure levels were measured to calculate the cumulative noise exposure (CNE), and an audiometric test was performed by an experienced technician in a soundproof booth. Sex differences in and influencing factors of low-frequency (LFHL) and high-frequency hearing loss (HFHL) were analyzed using logistic regression models stratified by age and CNE.

**Results:**

At comparable noise exposure levels and ages, the prevalence of HFHL was significantly higher in males (34.4%) than in females (13.8%), and males had a higher prevalence of HFHL (OR = 4.19, 95% CI 3.18 to 5.52) after adjusting for age, CNE, and other covariates. Sex differences were constant and highly remarkable among subjects aged 30 to 40 years and those with a CNE of 80 to 95 dB(A). Alcohol consumption might be a risk factor for HFHL in females (OR = 3.12, 95% CI 1.10 to 8.89).

**Conclusions:**

This study indicates significant sex differences in NIHL. Males are at higher risk of HFHL than females despite equivalent noise exposure and age. The risk factors for NIHL might be different in males and females.

## Introduction

Noise-induced hearing loss (NIHL) is one of the most common occupational diseases worldwide [1]; it results in a considerable economic burden and personal physiological impacts, especially among those in developing countries [2]. NIHL is mainly caused by loud sound damage and is probably influenced by age, sex, genetics, underlying diseases, personal behaviors, and other physical and chemical hazards [3]. These factors contributing to individual differences in susceptibility to NIHL have long been of interest.

The existence of sex differences in NIHL in humans has been disputed. This is an important issue since exposure to various occupational noises has become increasingly prevalent in females recently [4]. Several recent studies have indicated that working in preschools and maternity wards might increase the risk of hearing loss and tinnitus in females [5, 6]. However, relatively little information is known about the epidemiologic features, audiological characteristics, and influencing factors of occupational hearing loss in females. Although many researchers have pointed out that it is necessary to consider sex an explanatory variable in occupational health studies [7], the vast majority of occupational noise exposure studies included few females, and some even focused on only males [8–11]; this might partly be due to the high proportion of males among noise-exposed workers [12].

Several large-scale epidemiological surveys that included a number of females noted that occupational NIHL seems to be more prevalent in males than in females [13–15]; nevertheless, there are some concerns that still require exploration, such as whether the sex differences associated with NIHL were actually caused by the differences in noise exposure levels or behavioral habits between males and females. In fact, some animal studies have suggested that sex might be an internal biological factor that affects the characteristics and therapeutics of NIHL; thus, this factor should be considered in NIHL studies [16, 17]. A precisely controlled noise exposure study in mice indicated that females were protected from NIHL to a greater degree than males, while otoprotection provided by the therapeutic suberoylanilide hydroxamic acid (SAHA) was limited to males [16]. This evidence highlights the importance of exploring sex differences in NIHL in humans to optimize individual prevention and treatment strategies.

In the current study, hearing loss was assessed in 2280 shipyard industrial noise-exposed Chinese workers aged 18–60 years; the participants included 1140 males and 1140 females matched by age, job type, and employment length. Data on body mass index (BMI), noise exposure level, hearing protective device (HPD) usage, community noise, personal earphone usage, smoking, and alcohol consumption were collected to identify true sex differences in NIHL and other influencing factors.

## Methods

### Study population and design

This cross-sectional study included 1140 noise-exposed males and 1140 noise-exposed females enrolled at a shipyard in eastern China from August to October 2018. The study was performed in accordance with the principles stated in the Declaration of Helsinki and approved by the Ethics Committee of the Ninth People’s Hospital affiliated to Shanghai Jiao Tong University School of Medicine. All the participants signed informed consent forms.

Recruitment notices were sent to workers in our cooperative shipyard with the help of administrative staffs. After collection of demographic characteristics, information of occupation and hearing related symptoms of subjects by the face-to-face investigation, male and female subjects were matched in 1:1 ratio, according to the following criteria: (1) subjects had an age difference of less than 2 years; (2) subjects had the same job type with comparable task assignments in the same department; and (3) subjects had the same cumulative time of noise-exposure. Subjects then underwent the electro-otoscopy, tympanometry, and air-conduction pure tone audiometry (see the detailed methodology below).

The inclusion criteria were as follows: (1) Chinese Han ethnic population (China’s main nationality) aged 18–60 years; (2) no history of otological traumas, surgeries or diseases, ototoxic drug use, or a family history of hearing loss; (3) no history of chronic diseases (such as hypertension, diabetes, and abnormal hepatorenal function) requiring treatment; (4) a history of work in a single type of job with exposure to industrial noise, with a work-day equivalent A-weighted sound pressure level of at least 80 dB(A) for a continuous 8 h (*L*_*Aeq,8h*_); (5) no exposure to gun shots or bombs.

In this study, 1218 pairs of male and female subjects (a total of 2436) were initially recruited, and 1140 pairs (a total of 2280 subjects) from which were included finally.

### Noise exposure level assessment

Work-day individual *L*_*Aeq,8h*_ was assessed using a wearable personal exposure dosimeter (Aihua, ASV5910 type, Hangzhou, China) in accordance with the standards of IEC 61672:2002 and IEC 61252:2002. Subjects were asked to wear the dosimeter on their right shoulder from 8:00 am to 4:00 pm on regular work days. Cumulative noise exposure (CNE) was used to estimate the effects of noise exposure for each subject. Since all the subjects included in this study had stable job types, the CNE was calculated using the following formula [18]:

**CNE =** ***L***_**Aeq,8*h***_ **+ 10log*T***

where *T* is the occupational noise exposure time-length in years.

### Auditory examination

Audiometry was performed by an experienced audiological technician using an audiometer with TDH-39P headphones (Otometrics Madsen, Xeta, Denmark) in a soundproof booth with background noise below 25 dB(A). Pure tone air-conduction audiometric thresholds for both ears at frequencies of 0.5, 1, 2, 3, 4, 6 and 8 kHz were measured in 5-dB steps in accordance with the regulations of ISO 8253-1:2010. Subjects were asked to avoid exposure to occupational noise or loud sounds within 16 h before the hearing examination.

### Covariates

In addition to data on demographic characteristics such as biological sex (collected from the identity card), age (years), BMI (categorized as normal, overweight, and obese according to the Centers for Disease Control and Prevention guidelines) and occupational noise exposure time (years), data on individual health-related behavioral covariates were collected during face-to-face interviews; health-related data included (1) community noise exposure (louder than normal conversations were marked as “yes”); (2) individual habits that might affect hearing status, such as HPD usage (at least 4 h per weekday was marked as “yes”), personal earphone usage (at least 2 h per day was marked as “yes”), smoking (at least 10 cigarettes per day was marked as “yes”) and alcohol consumption (at least 50 g of alcohol per day was marked as “yes”); and (3) self-reported auditory symptoms, such as hearing difficulty (never, sometimes, or often) and tinnitus.

In the current study, we calculated the average low-frequency (0.5, 1, and 2 kHz) hearing threshold and high-frequency (3, 4, 6, and 8 kHz) hearing threshold of the better ear, and hearing loss was defined as an average hearing threshold > 25 dB HL.

### Statistical analysis

Continuous variables are expressed as means ± SDs, and categorical variables are presented as percentages (%). Normality tests of continuous variables were conducted using kurtosis and skewness coefficients. Between males and females, statistical significance for differences in continuous variables was examined using Student’s *t* test or the Mann-Whitney *U* test, and categorical variables were compared by the chi-square test. The association between hearing thresholds and age or CNE was examined with linear regression analysis, and a binary logistic regression model was constructed to analyze the possible influencing factors of hearing loss. Sex, age, and CNE groups were stratified in the analyses. A *P* value < 0.05 was considered statistically significant. Data analyses were performed using SPSS software version 25.0 (IBM Corporation, Armonk, NY, USA).

## Results

### Basal characteristics of male and female workers

The characteristics of the 1140 male subjects and 1140 female subjects are presented in Table 1. The mean age of the subjects was 35.0 ± 8.4 years, ranging from 18 to 60 years, and the mean CNE was 92.9 ± 8.8 dB(A), ranging from 80.0 to 117.2 dB(A). Due to the strict matching design in this study, there were no statistically significant differences in age or CNE between male and female workers. Approximately 7.2% (165/2280) of the workers had low-frequency hearing loss (LFHL), and 24.0% (548/2280) had HFHL. The prevalence rates of LFHL and HFHL in males were significantly higher than those in females. Regarding hearing health-related behaviors, males reported more community noise exposure and personal earphone usage, while females reported more HPD usage. In addition, males had higher BMI and rates of alcohol consumption and smoking than females.

**Table 1:** Characteristics of male and female workers exposed to industrial noise (total *n* = 2280)

Variables	Males (***n*** = 1140)	Females (***n*** = 1140)	***P*** value
**Age, mean ± SD**	35.0 ± 8.4	35.0 ± 8.4	0.996
**Cumulative noise exposure, mean ± SD**	92.9 ± 8.8	92.9 ± 8.8	0.920
**Body mass index,** ***n*** **(%)**			< 0.001
Normal	643 (56.4)	758 (66.5)	
Overweight	374 (32.8)	268 (23.5)	
Obese	83 (7.3)	38 (3.3)	
**Hearing protective device use,** ***n*** **(%)**			0.028
< 4 h per day	462 (40.5)	411 (36.1)	
>= 4 h per day	678 (59.5)	729 (63.9)	
**Community noise exposure,** ***n*** **(%)**			< 0.001
No	714 (62.6)	820 (71.9)	
Yes	426 (37.4)	320 (28.1)	
**Personal earphone use,** ***n*** **(%)**			0.001
< 2 h per day	830 (72.8)	897 (78.7)	
>= 2 h per day	310 (27.2)	243 (21.3)	
**Smoking,** ***n*** **(%)**			< 0.001
< 10 cigarettes per day	674 (59.1)	1132 (99.3)	
>= 10 cigarettes per day	466 (40.9)	8 (0.7)	
**Alcohol consumption, n (%)**			< 0.001
< 50 g alcohol per day	793 (69.6)	1116 (97.9)	
>= 50 g alcohol per day	347 (30.4)	24 (2.1)	
**Hearing difficulty,** ***n*** **(%)**			0.780
Never	441 (38.7)	439 (38.5)	
Sometimes	472 (41.4)	461 (40.4)	
Often	227 (19.9)	240 (21.1)	
**Tinnitus,** ***n*** **(%)**			0.307
No	804 (70.5)	827 (72.5)	
Yes	336 (29.5)	313 (27.5)	
**Low-frequency hearing loss,** ***n*** **(%)**			0.010
No	1041 (91.3)	1074 (94.2)	
Yes	99 (8.7)	66 (5.8)	
**High-frequency hearing loss,** ***n*** **(%)**			< 0.001
No	749 (65.7)	983 (86.2)	
Yes	391 (34.3)	157 (13.8)	

Males and females were initially matched for age and noise exposure level

### Notched audiograms of male and female workers

**Fig. 1:**
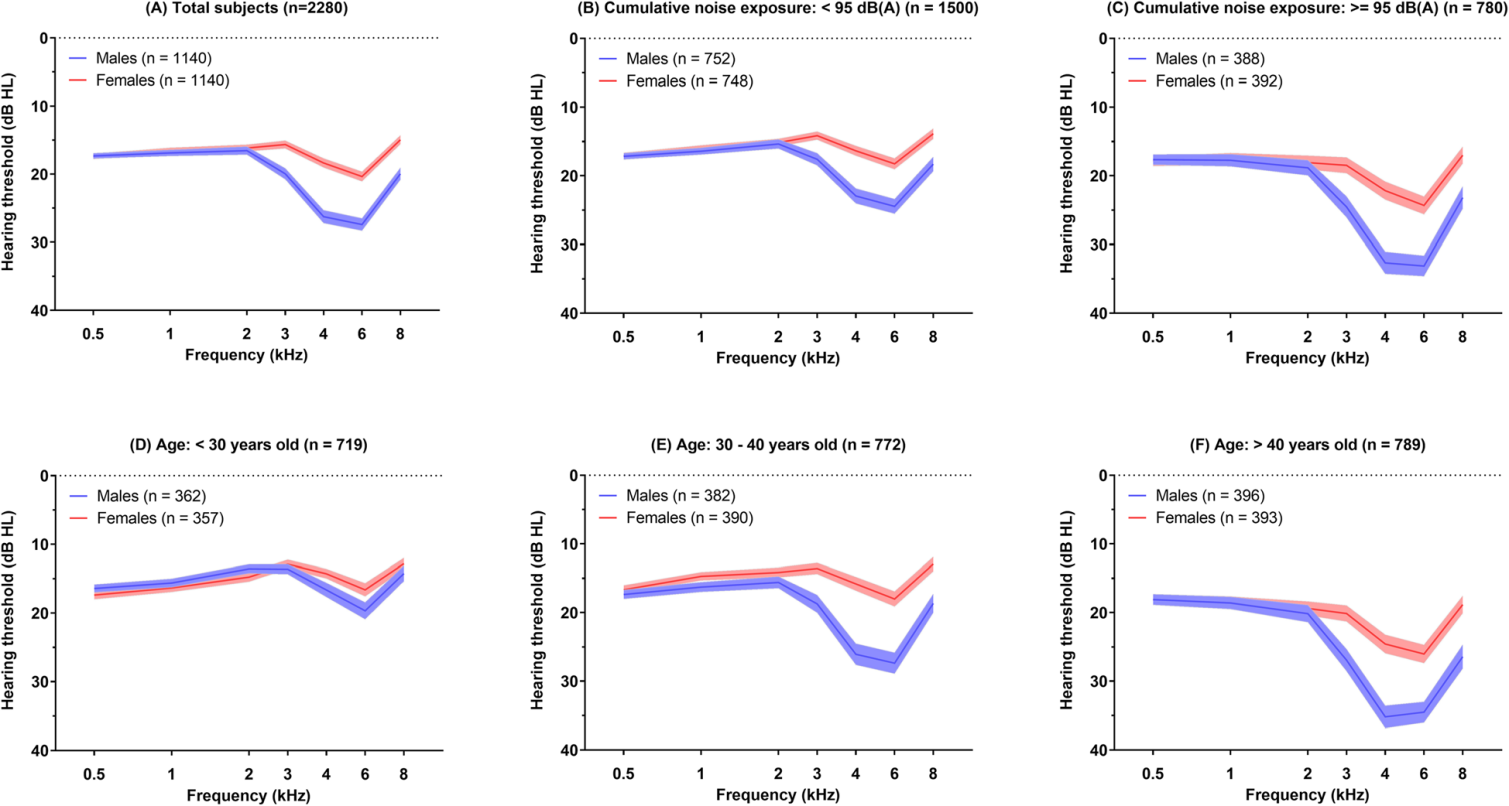
Stratified description of audiogram characteristics between males and females. Average hearing thresholds are shown at each frequency of 0.5, 1, 2, 3, 4, 6, and 8 kHz with 95% confidence intervals

To preliminarily explore detailed sex-specific differences and characteristics of NIHL, the average audiogram results of each ear in males and females were analyzed. Since noise exposure and age are well-known influencing factors of hearing status, subjects were stratified according to CNE and age. As shown in Fig. 1a, average audiogram results showed high-frequency (3, 4, 6, and 8 kHz) notched hearing threshold shifts regardless of sex, representing typical noise-induced hearing impairment. Considering the high-frequency threshold notch, the differences in notch depths between males and females were larger in the subgroups with greater noise exposure (Fig. 1b, c) and advanced age (Fig. 1d–f). In comparison, sex differences in hearing thresholds at low frequencies were relatively minor.

### Sex differences associated with noise, age, and hearing loss

**Fig. 2:**
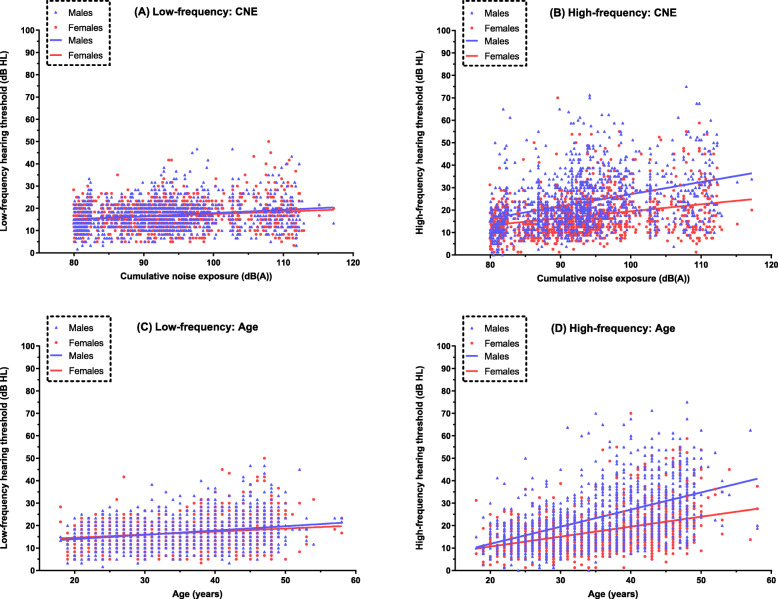
Scatter plots of mean hearing thresholds by cumulative noise exposure and age. Plots are stratified by sex, and the lines represent the trends of the linear associations

Figure 2 shows the linear associations of hearing loss with CNE and age in males and females. Mean low-frequency hearing thresholds were associated with a per unit increase in CNE of 14.8% dB HL in males (95% CI 0.15 to 0.19, *P* < 0.001) and 11.2% dB HL in females (95% CI 0.07 to 0.15, *P* < 0.001), as well as with a per year age increase of 19.2% dB HL in males (95% CI 0.15 to 0.23) and 13.6% dB HL in females (95% CI 0.09 to 0.18). However, the sex differences were nonsignificant in associations of low-frequency hearing thresholds with CNE (Fig. 2a, *P* = 0.065) or age (Fig. 2c, *P* = 0.230), according to the linear slopes of the trends.

In contrast, males and females had remarkably significantly different linear associations of high-frequency hearing thresholds with CNE (Fig. 2b, *P* < 0.001) and age (Fig. 2d, *P* < 0.001). Mean high-frequency hearing thresholds were associated with a per unit increase in CNE of 54.1% dB HL in males (95% CI 0.46 to 0.62, *P* < 0.001) but only 31.0% dB HL in females (95% CI 0.25 to 0.37, *P* < 0.001), as well as a per year age increase of 76.4% dB HL in males (95% CI 0.69 to 0.84) and 44.4% dB HL in females (95% CI 0.39 to 0.50).

### Different influencing factors of hearing loss in males and females

Among all the subjects, the variables of sex, age, CNE, BMI, HPD usage, community noise exposure, personal earphone usage, smoking, and alcohol consumption were included in the logistic regression analyses of LFHL and HFHL. All the variables noted above except sex were further separately analyzed in males and females to explore sex differences in the influencing factors of hearing loss. Table 2 shows the logistic regression results. Males had a significantly higher risk of both HFHL (OR = 4.19, 95% CI 3.18 to 5.52) and LFHL (OR = 1.49, 95% CI 1.00 to 2.20) after adjustments for other covariates. As expected, age and CNE were significant influencing factors of HFHL and LFHL in both males and females. Notably, alcohol consumption was regarded as a risk factor for HFHL in all subjects (OR = 1.36, 95% CI 1.01 to 1.83); however, a significant association between alcohol consumption and hearing loss was shown in only females (OR = 3.12, 95% CI 1.10 to 8.89).

**Table 2:** Influencing factors of high-frequency and low-frequency hearing loss in in the total population, males, and females

	Total (***n*** = 2280) OR (95% CI)	Males (***n*** = 1140) OR (95% CI)	Females (***n*** = 1140) OR (95% CI)
**High-frequency hearing loss**			
**Sex**			
Females	Reference	/	/
Males	4.19 (3.18–5.52)	/	/
**Age, years**	1.13 (1.12–1.15)	1.13 (1.11–1.16)	1.14 (1.11–1.18)
**Cumulative noise exposure, dB(A)**	1.04 (1.03–1.06)	1.04 (1.02–1.06)	1.04 (1.02–1.06)
**Alcohol consumption**			
< 50 g alcohol per day	Reference	Reference	Reference
>= 50 g alcohol per day	1.36 (1.01–1.83)	1.28 (0.94–1.74)	3.12 (1.10–8.89)
**Low-frequency hearing loss**			
**Sex**			
Females	Reference	/	/
Males	1.49 (1.00–2.20)	/	/
**Age, years**	1.11 (1.08–1.13)	1.11 (1.07–1.15)	1.10 (1.06–1.15)
**Cumulative noise exposure, dB(A)**	1.04 (1.02–1.06)	1.03 (1.01–1.06)	1.06 (1.02–1.09)

*OR* odds ratio, *CI* 95% confidence interval

Variables without statistical significance in the regression models are not shown

### Sex differences in NIHL

**Fig. 3:**
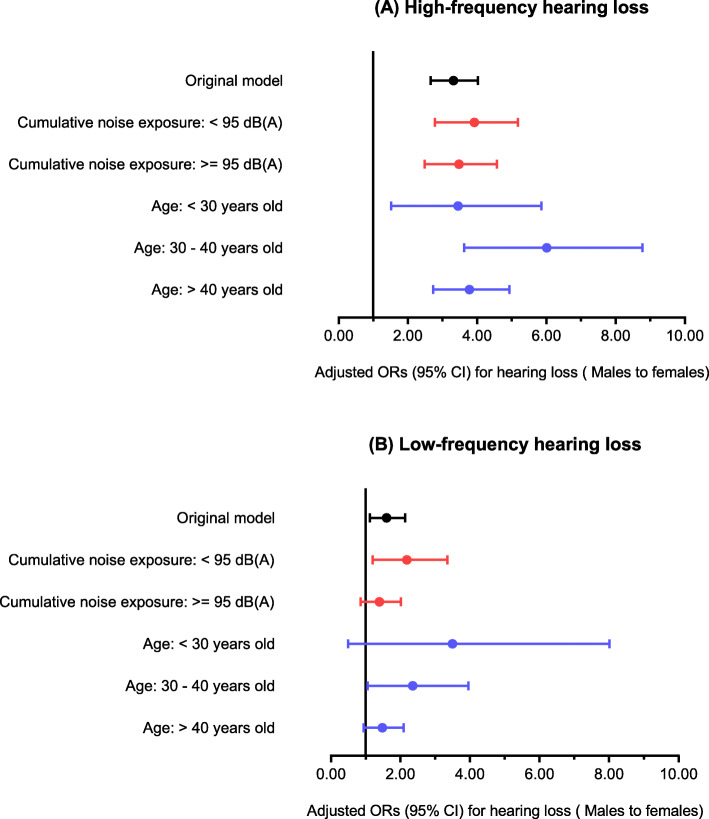
Stratified regression analysis considering cumulative noise exposure and age. The association between sex and HFHL (**a**) and LFHL (**b**)

Since age and CNE were significant covariates of hearing loss, sex differences were further analyzed considering age and noise exposure level stratification. Subjects were stratified into the following groups: CNE < 95 dB(A) and >=95 dB(A) and age < 30, 30–40, and > 40 years old. Adjusted ORs with 95% CIs for sex differences (males and females) in hearing loss among each subgroup of subjects are shown in Fig. 3. According to the stratified regression models, though slight differences in ORs were observed among different subgroups, male sex was still a remarkable risk factor for HFHL. In contrast, sex was not always significantly associated with LFHL, and the ORs for LFHL according to sex in the original model were lower than those for HFHL.

## Discussion

In general, the present study revealed that under comparable loud occupational (industrial) noise exposure conditions, HFHL was significantly more prevalent in males than females after adjustments for age, noise exposure level, and other potential behavioral confounding factors. Notably, we found that alcohol consumption was a covariate associated with HFHL in only females; this association was nonsignificant in males. Overall, our study results suggested that males and females have different responses to noise exposure damage, in accordance with some findings in previous animal studies [16, 17, 19].

### Sex bias in NIHL studies

In the current study, we recruited a relatively large number of females (*n* = 1140) with precise individual noise exposure level measurements and audiograms. To date, the epidemiological characteristics and audiological outcome of occupational noise-induced hearing impairment in females are still unclear in comparison with males. Most studies had to marginalize the sex differences mainly due to the lack of enough female subjects for analyses. For instance, a Norwegian study assessed the risk of NIHL by audiometric notches among 4627 train and track maintenance male workers [8]. A study of 790 aircraft-manufacturing workers in Taiwan showed that 27.1% of them had high-frequency hearing loss [20], while another recent study suggested that 10.3% of 1214 Chinese male train drivers had HFHL. Even a large-scale study of NIHL among 12055 Norwegian railway workers could include only approximately 680 noise-exposed female workers since the proportion of females in the noise-exposed workforce is usually less than 10% [13]. According to the Nord-Trøndelag Hearing Loss Study (NTHLS), which included 49,774 subjects, there were fewer women who reported high noise exposure (334/26477) than men (4566/23297 )[21]. In addition, there are some recent small-scale studies focused on females, for instance, a cross-sectional study including 115 female employees in obstetric wards and a cohort study among preschool teachers in Sweden focused on sound-induced auditory fatigue, while the limitation might be using self-reported hearing-related symptoms as the main outcome measures [5, 6].

### Sex bias in noise exposure level

Further, to assess sex differences in NIHL, we matched males and females with equivalent noise exposure levels and ages, which were the main influencing factors of hearing loss (Table 1). In this study, the percentage of HFHL in males (34.4%) was more than twice that in females (13.8%); this result was in accordance with many previous investigations which found that males have a higher prevalence of hearing loss than females. The National Institute for Occupational Safety and Health (NIOSH) Occupational Hearing Loss Surveillance Project, which collected millions of audiograms of U.S. workers (78% were males and 22% were females), showed a greater percentage of hearing impairment in males (14%) than in females (7%) [14]. However, as mentioned in many studies, it remains unclear whether the higher prevalence of hearing loss in males is partly because males are more likely to be exposed to hazardous workplace noise than females [22]. Indeed, most previous studies assessed noise exposure levels using self-reported information from workers without thorough noise exposure measurements in noisy environments [21]. However, there have been some questionnaires and tools that have been shown to be beneficial for occupational hazard research to estimate the self-reported average 8-h noise exposure level, such as the job-exposure matrix (JEM) for occupational noise [23], which has been validated in several epidemiological studies. However, the validity of self-reported occupational noise exposure might be limited because of the different work hours, task assignments, and workplace conditions between males and females, even within the same occupation [12]. Thus, we matched males and females with comparable task assignments in the same work department to minimize differences in noise exposure levels, and the results of noise exposure level measurements were equivalent as expected.

### Female-specific protective effects in NIHL

It has been broadly accepted that the first sign of NIHL is a “notch” of the audiogram at high frequencies of 3, 4, or 6 kHz, with recovery at 8 kHz, and the average thresholds at 0.5, 1, and 2 kHz are usually better than those at high frequencies [1]. However, we did not assess the prevalence of “notch” audiogram in this study since there is still lacked of specific, objective criteria for detecting an audiometric notch according to any international standard or guidance [24]. Instead, we calculated the low-frequency and high-frequency hearing thresholds and found that the sex differences in hearing loss were constantly significant at high frequencies but not at low frequencies after adjustments for each potential confounding factor, suggesting internal biological differences between males and females in high-frequency specific hearing damage from noise exposure.

According to the regression models for HFHL, aging and CNE level were the main risk factors in both males and females (Table 2), which was in accordance with previous studies [14, 21]. Nevertheless, the interactions between noise exposure, aging, and sex in hearing loss are complicated. Therefore, we analyzed the ORs for hearing loss by sex in subgroups stratified by noise exposure and age. Our results suggested that the sex differences were remarkable, especially among workers aged 30 to 40 years and those with a CNE of 80 to 95 dB(A) (Fig. 3). It has been shown that hearing sensitivity declines faster in males than in females at most ages and frequencies [25, 26], but in older populations, females might have a significantly faster rate of high-frequency hearing threshold changes than males [27]. The effects of noise damage and aging coexisting in the same ear remain unclear [28]. According to new findings on noise-induced hidden hearing loss and cochlear synaptopathy in recent years [29], some portion of age-related hearing loss probably arises from lifetime accumulated noise exposure [30]. Thus, we speculated that the sex-specific protective effects on hearing loss might be weakened by increased noise damage and aging. Besides, in younger workers, perhaps the sex differences were underestimated due to the relatively less noise exposure level and existence of hidden hearing loss, which presents as normal hearing in audiometry.

### Consideration of confounding factors and sex differences

In addition to noise exposure and aging, many previous studies have indicated that genetic factors, personal factors (education, income, leisure-time noise, HPD usage, smoking, alcohol consumption, diseases and drug usage), occupational environmental exposures other than noise (solvents, heavy metals, carbon monoxide (CO), vibration, heat) and shiftwork might influence the risk of NIHL [3]. In this study, male and female subjects were matched by job and department, and it is reasonable to assume that the conditions of education, income, other occupational environmental exposures and shiftwork were comparable. The effects of otological diseases, some chronic diseases and related drug therapies were avoided by applying the inclusion criteria. Detailed information on leisure-time noise exposure, HPD usage, smoking and alcohol consumption was collected via a face-to-face interview with a questionnaire (Table 1).

With consideration of those confounding factors, our results showed that in only female workers, consuming at least 50 g of alcohol per day might be a risk factor for HFHL. There are disparate findings on whether alcohol consumption increases the risk of hearing loss [31]. A large UK population-based study including 164,770 adults reported that those who consumed alcohol were less likely to experience hearing loss than lifetime teetotalers, suggesting that alcohol consumption had a protective effect [32]. In contrast, the Nurses’ Health Study II (NHS II) reported no significant association between total alcohol consumption and risk of hearing loss [33]. Further, larger prospective cohort studies are still needed, and the associations need to be analyzed among males and females separately.

### Underlying mechanism: estrogen makes a difference?

With the increasing understanding of sex differences in the function and regulation of the auditory system [34, 35], several underlying mechanisms have been proposed, and the main studies have focused on estrogen signaling. Substantial evidence has linked levels of estrogen to auditory function in various human population and animal studies [36]. Several animal studies have provided evidence supporting the protective potential of estrogen in noise-induced cochlear dysfunction. Noise exposure caused greater auditory function damage in ovariectomized rats with estrogen deficiency than normal rats, as demonstrated by decreased distortion-product otoacoustic emissions (DPOAEs) and auditory-evoked brainstem response (ABRs) [37]. The commonly used anti-estrogenic agent tamoxifen was also reported to promote noise exposure-related physiological damage affecting cochlear compound action potentials (CAPs) and DPOAEs in gerbils [38]. The audiological effects of estrogens might be mediated by estrogen receptor beta (ERβ; also known as ESR2). ERβ-knockout mice are more susceptible to acoustic trauma than wild-type and ERα-knockout mice, manifested as temporary increment of ABR thresholds [39]. Our results suggested the obviously increased HFHL (Fig. 1) and reduced sex-specific otoprotection (Fig. 3) among female workers aged older than 40 years, whom were probably accompanied by decreased level of estrogen. Although it is still not clear whether NIHL is associated with circulating estrogen levels in humans, there is the possibility that the sex differences in NIHL shown in animal studies could present similarly in humans.

### Strengths and limitations

The primary strength of our study is the inclusion of a relatively large number of occupationally noise-exposed females who were well-matched with males. To our knowledge, this is the first study to focus on sex differences in NIHL in humans by assessing individual noise exposure, audiogram results and detailed information regarding probable confounding factors. One limitation of the study might be the possibility of genetic differences existing among subjects, although we have taken the family history of hearing loss into consideration. Another limitation is that the potential impact of subjects’ cumulative lifetime noise exposure on study results. We only matched for the occupational noise exposure, while the leisure-time noise exposure might be quite different between males and females since their different roles in human society. In addition, we used only the current audiogram results in the association analyses because of the lack of baseline data; however, all the subjects had to demonstrate normal hearing to pass the entry audiometry assessment before being offered the job. A longitudinal study of sex differences in NIHL still needs to be conducted.

## Perspectives and significance

In summary, previous studies have proved the significant sex differences in auditory system, while few studies investigated sex differences in NIHL, and the sex bias in human studies resulted in inadequate data from females. Here we conducted a study included relatively large-scale occupational noise-exposed females with well-matched males to provide the population-based evidence of sex differences in NIHL. Through assessment of the exact individual noise exposure, audiogram and detailed information on many probable confounding factors, we demonstrated that even under comparable noise exposure conditions, females experienced less HFHL than males. In addition, the risk factors for NIHL might be different in males and females. Our findings indicate that sex differences should be considered, and data should be stratified by sex when conducting risk association studies and therapeutic studies of NIHL in the future.

## Data Availability

All data generated or analyzed during this study are included in this published article.
